# P-1777. Low adherence to institutional antibiotic clinical practice guideline in patients diagnosed with *Clostridioides difficile* infection

**DOI:** 10.1093/ofid/ofae631.1940

**Published:** 2025-01-29

**Authors:** Joseph Marcus, Kayla R Scheps, Zachary K Matthews

**Affiliations:** Brooke Army Medical Center, San Antonio, TX; Brooke Army Medical Center, San Antonio, TX; San Antonio Uniformed Services Health Education Consortium, San Antonio, Texas

## Abstract

**Background:**

Previous antimicrobial therapy is a common risk factor for *Clostridioides difficile* infections (*CDIs*). Clinical practice guidelines (CPGs) assist clinicians in prescribing the correct antibiotics for various clinical syndromes to avoid unnecessary or overly broad-spectrum antibiotic use. Retrospective analysis of *CDIs* may serve as a guide for the high-risk antimicrobials included in CPG development.

**Figure:**
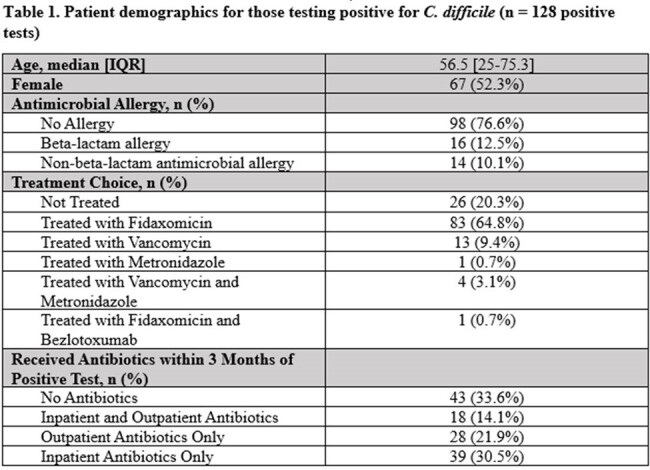

**Methods:**

Patients who tested positive for *C. difficile* at Brooke Army Medical Center from November 2022 to October 2023 were reviewed. Demographics, *C. difficile* treatment, antibiotic allergies, antibiotics received in the preceding 90 days prior to a positive test, and indication for antibiotic therapy were recorded. If a treatment indication appeared on the institutional CPG, adherence to the antibiotic recommendation was recorded. Patients who received no antibiotics or only antibiotics as guided by the correct CPG were considered unpreventable.

**Figure:**
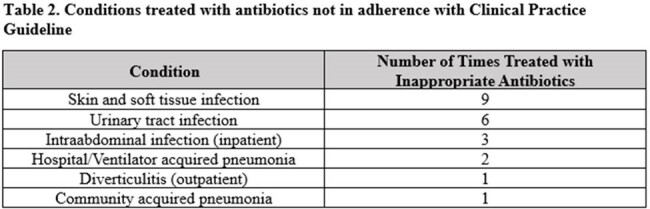

**Results:**

During the study period, 128 positive cases were identified. The median patient age was 56.5 years [IQR: 25-75.3] (Table 1). 80% of cases were ultimately treated for infection, with fidaxomicin representing the treatment of choice in 81% of treated patients. In the preceding 3 months prior to a positive test, 67% of patients had at least one day of antibiotic exposure. The median number [IQR] of total antibiotic days within 30, 60, and 90 days of a positive test was 1 [0-7], 3.5 [0-8.75], and 5 [0-9.75] respectively. For 47 (55%) cases, all antibiotic indications within the previous 90 days appeared on the CPG. However, prescribers did not adhere to the CPG (Table 2) in 22 (47%) of these cases. The most common inpatient and outpatient indications not on the CPG were perioperative antibiotics and otitis media, respectively. Overall, only 58 cases (45%) were considered unpreventable.

**Conclusion:**

In this single center study, retrospective analysis of individual *C. difficile* cases provided direction for the antimicrobial stewardship team by identifying CPG recommendations that were not followed as well as identifying conditions not on previous CPGs. Tying patient harm from *CDIs* with inappropriate antimicrobial use builds the local evidence base to prevent future *CDIs*.

**Disclosures:**

**All Authors**: No reported disclosures

